# Supplementary material for: Biomolecular condensate drives polymerization and bundling of the bacterial tubulin FtsZ to regulate cell division
Source: Nat Commun. 2023 Jun 28;14:3825. doi: 10.1038/s41467-023-39513-2 (PMC10307791; doi:10.1038/s41467-023-39513-2)
Supplement: Supplementary file 1 — Supplementary Information [file 41467_2023_39513_MOESM1_ESM.pdf]

Supplementary Information for

Biomolecular condensate drives polymerization and bundling of the bacterial tubulin FtsZ to regulate cell division

Beatrice Ramm, Dominik Schumacher, Andrea Harms, Tamara Heermann, Philipp Klos, Franziska Müller, Petra Schwille and Lotte Søgaard-Andersen

This file contains

- Supplementary Figure 1-14
- Supplementary Tables 1-3
- Supplementary References

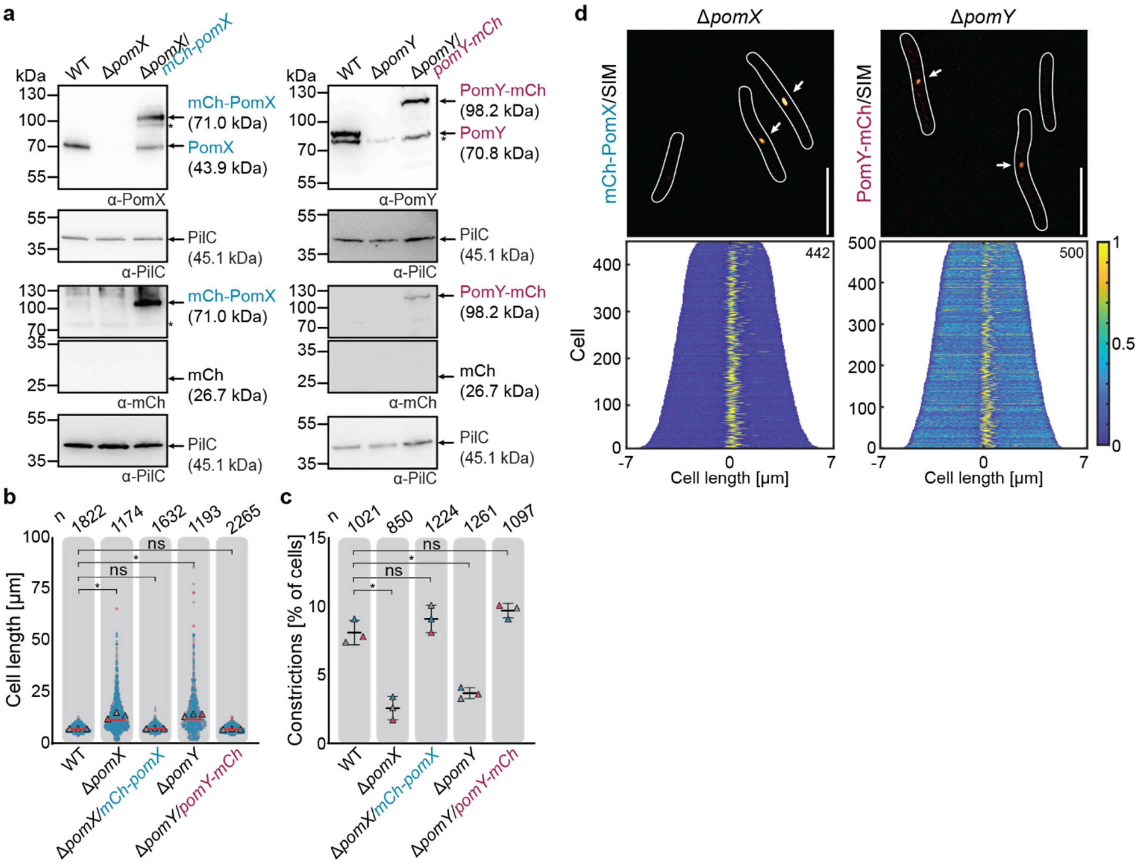

Supplementary Figure 1. Native level expression of mCh-PomX and PomY-mCh restores a WT phenotype.

a. Immunoblot analysis of mCh-PomX and PomY-mCh accumulation. The same amount of protein was loaded per lane, separated by SDS-PAGE, blotted, and probed with α-PomX, α-PomY, α-mCh and α-PilC (loading control) antibodies. Molecular size markers are indicated on the left. * indicates unspecific binding of the antibodies used. Proteins with their predicted MW are indicated on the right. Experiments were repeated three times with similar results, and representative data are shown.

b. Cell length distribution of indicated strains. Data from three independent replicates are shown as magenta, grey, and teal. The mean for each experiment is shown as triangles in the relevant color. Red line indicates the median. The number of analyzed cells (n) is shown at the top. * indicates significant difference; ns, not significant, in a 2way ANOVA with multiple comparisons to WT. *P*-values from left to right $P<0.0001$, $P=0.9983$, $P<0.0001$, $P=0.9997$.

c. Constrictions frequency analysis of strains in b. Data from three independent replicates are shown as magenta, grey, and teal with the mean±STDEV. * indicates significant difference; ns, not significant in 2way ANOVA with multiple comparisons to WT. *P*-values from left to right $P=0.0001$, $P=0.5348$, $P=0.0007$, $P=0.1685$.

d. Analysis of cells expressing mCh-PomX and PomY-mCh at native levels by SIM microscopy. White arrows point to mCh-PomX and PomY-mCh clusters. White lines indicate cell outlines. Scale bars, 5 µm. Demographs show fluorescence signals of cells sorted according to length and with off-center signals to the right. Number of cells (n) is indicated in the upper right. The colored scale bar indicates fluorescence signal intensity with the highest signal set to 1.

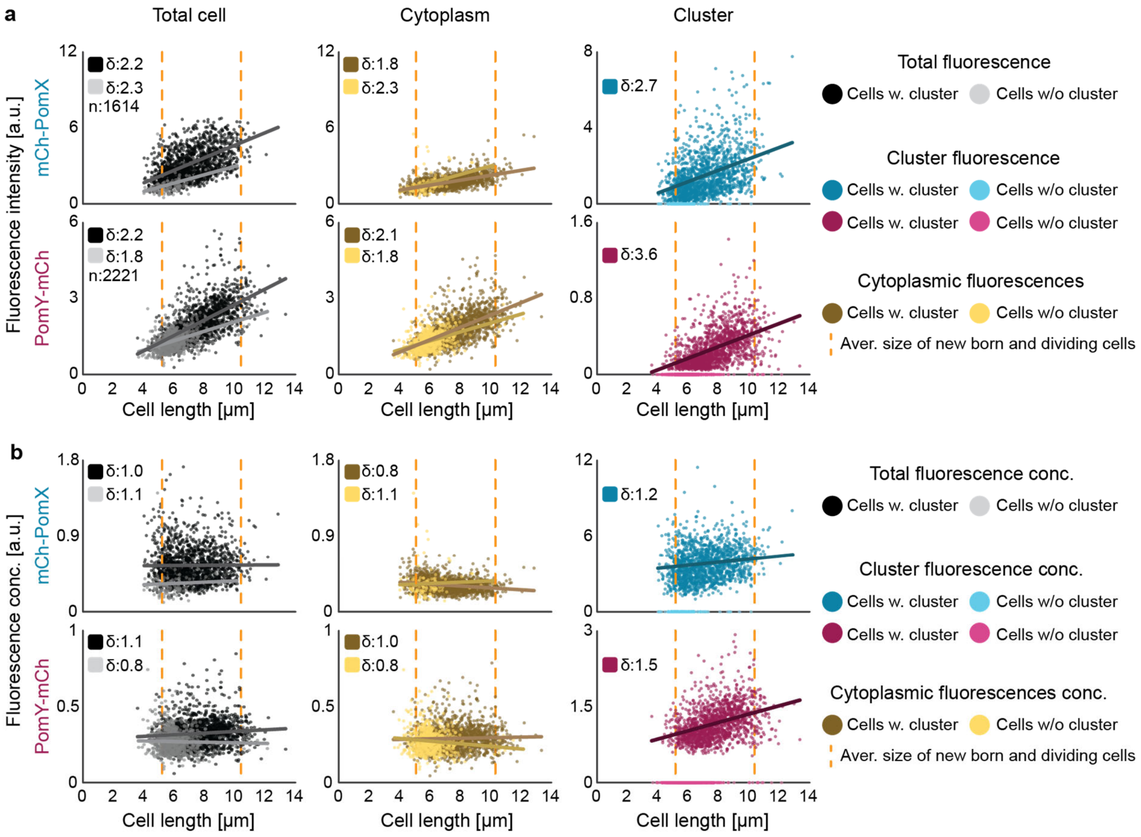

Supplementary Fig. 2. Population-based quantification of cellular, cytoplasmic, and cluster fluorescence intensity of mCh-PomX and PomY-mCh cells.

a. Total cellular, cluster, and cytoplasmic fluorescence intensity of cells expressing mCh-PomX (upper panels) and PomY-mCh (lower panels) at native levels as a function of cell length. Orange stippled lines indicate length of average newborn and dividing cell. Dark-colored lines are linear regression. δ values indicate the fold increase between birth and division. Data from three replicates are shown.

b. Total cellular, cluster, and cytoplasmic fluorescence concentrations of mCh-PomX (upper panels) and PomY-mCh (lower panels) in cells of a as a function of cell length. Graphs are as in a.

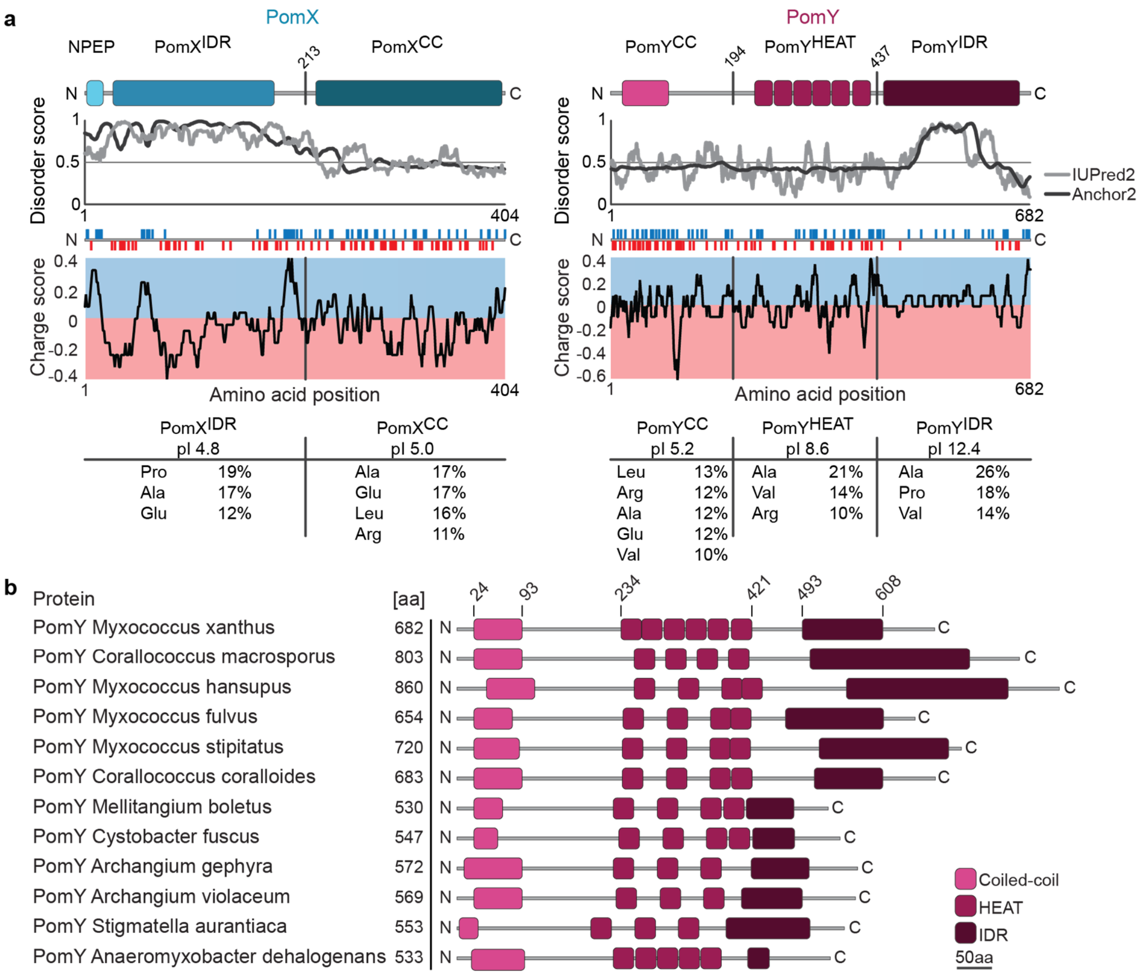

Supplementary Fig. 3. *In silico* characterization of PomX and PomY.
a. Domain architecture of PomX and PomY. PomX domain truncations used in[1] and PomY truncations as used here are indicated by vertical lines. $PomX^{IDR}$ extends from residue 1-213, $PomX^{CC}$ from 214-404, $PomY^{CC}$ from 1-194, $PomY^{HEAT}$ from 195-437, and $PomY^{IDR}$ from 438-682. Positively (Arg, Lys, His) and negatively charged (Glu, Asp) residues are indicated in red and blue. Charge score was plotted using a sliding window of six residues. Blue and red indicate positively and negatively charged regions, respectively. Amino acid composition is shown for individual domains for amino acids that make up >10% of residues.
b. Domain analysis of PomY orthologs. PomY orthologs were identified in a best-best hit reciprocal BlastP analysis from fully-sequenced genomes of myxobacteria as indicated on the left. Numbers on top indicate domain borders in PomY of *M. xanthus*.

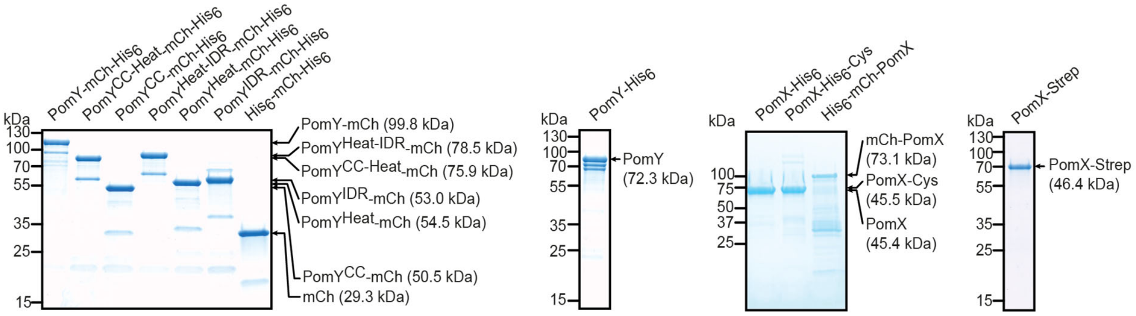

Supplementary Fig. 4. SDS-PAGE analysis of purified proteins.
Instant Blue stained SDS-PAGE analysis of purified PomY-mCh-His$_6$ variants, His$_6$-mCh, PomY-His$_6$ and PomX variants. Note that the His$_6$-tagged PomX and PomY variants are referred to as indicated on the right. Molecular size markers are shown on the left. 2 µg protein per lane were loaded. Note that PomY-mCh-His$_6$ does not separate to run at the calculated MW in SDS-PAGE, but instead runs at MW of ~120 kDa. Moreover, PomX-His$_6$, PomX-His$_6$-Cys, His$_6$-mCh-PomX and PomX-Strep also do not separate to run at the expected MW in SDS-PAGE, but instead run at MW of ~73 kDa, ~73 kDa, ~100 kDa and 70 kDa, respectively.

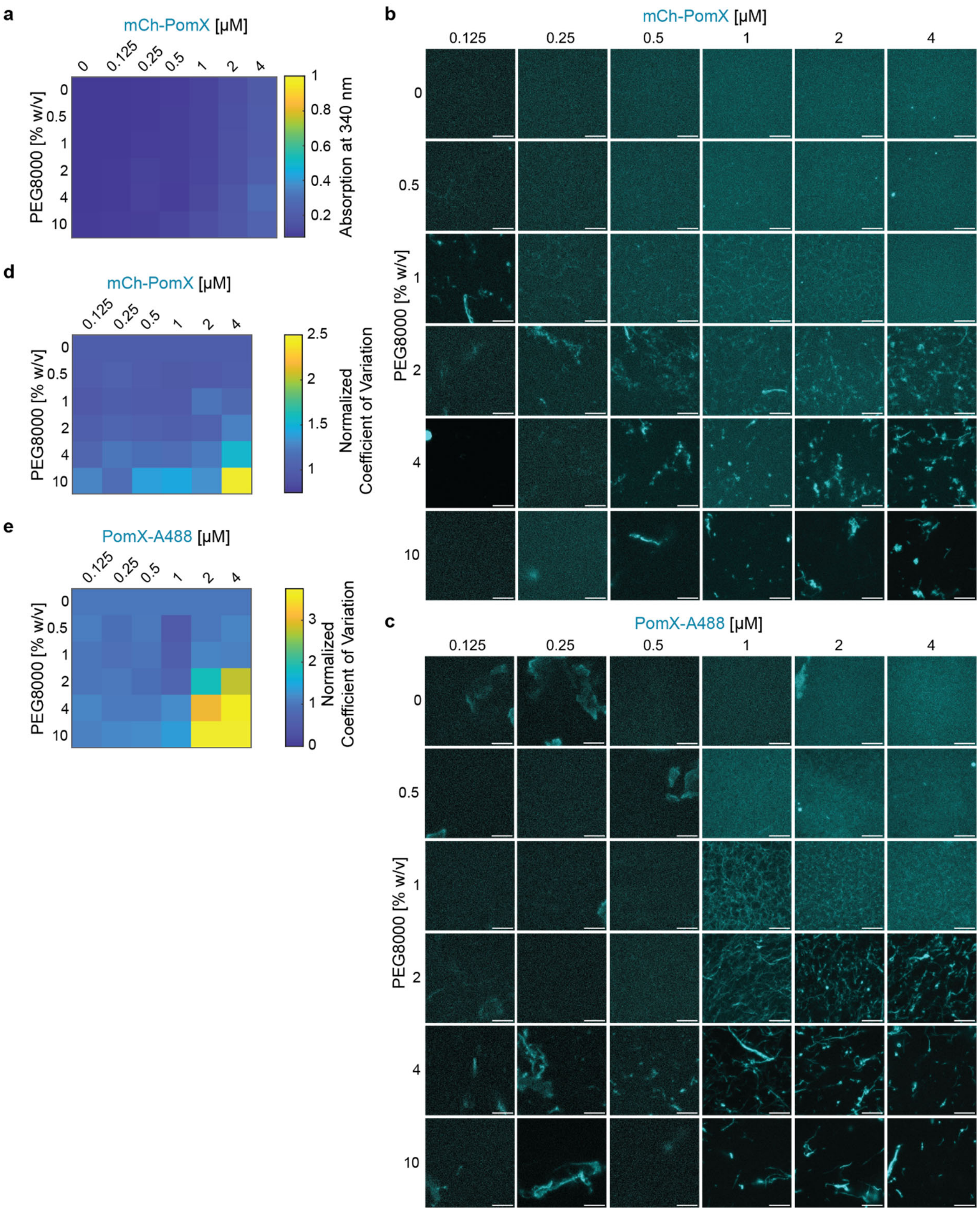

Supplementary Fig. 5. mCh-PomX and PomX form filaments in vitro that are bundled by PEG8000.
a. Turbidity measurements of mCh-PomX for increasing protein and PEG8000 concentrations. Heat map displays average absorption at 340 nm for n=3 replicates.
b, c. PomX forms filaments that are increasingly bundled with increasing concentrations of PEG8000. Representative maximum intensity projections of confocal z-stacks of mCh-PomX (b) and PomX-A488 (c) at different protein and PEG8000 concentrations. The images' brightness and contrast levels are not comparable but were individually adjusted for visualization purposes. Experiments were each performed once. Scale bars, 50 μm.

d, e. Heat map of the normalized CV of mCh-PomX (d) and PomX-A488 (e) for increasing protein and crowder concentrations. The CV was calculated as the STDEV over the mean of the fluorescence intensity. Values are the average coefficient of variation from the maximum intensity projection of confocal z-stacks from one representative experiment with n=4 (d) and n=2 (e) analyzed images per condition. All values obtained for each PomX concentration (0.125 - 4 uM) were normalized to the CV of the 0% PEG8000 condition for that concentration.

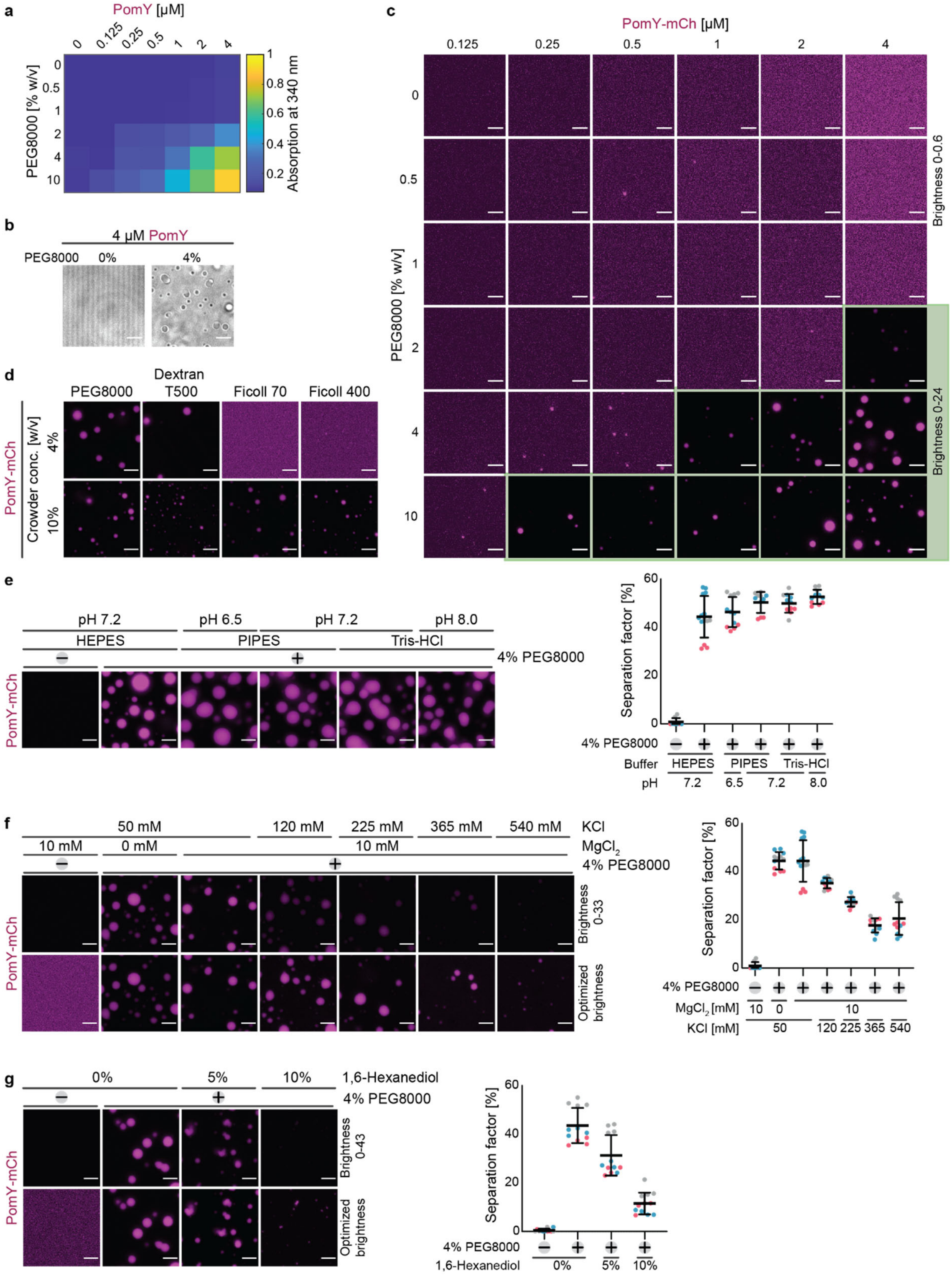

Supplementary Fig. 6. PomY and PomY-mCh phase separate in vitro.

a. Turbidity measurements of PomY for increasing protein and PEG8000 concentrations. The heat map displays average absorption at 340 nm for n=3 replicates.

b. Representative bright-field images of PomY in the absence and presence of PEG8000. Experiment was performed twice. Scale bars, 5 µm.

c. Representative images of PomY-mCh at different protein and PEG8000 concentrations. Brightness and contrast of the images were adjusted with two different thresholds for clarity indicated by a green background. Experiment was performed twice. Scale bars, 5 µm.
d. Representative images of 4 µM PomY-mCh in the presence of different artificial crowding agents. Experiment was performed three times. Scale bars, 5 µm.
e. Representative images of PomY-mCh in buffer with different pH based on different buffer substances (4 µM PomY-mCh, 0 or 4% PEG8000). The average separation factor (right panel) in percent is calculated from the maximum intensity projection of confocal z-stacks. Right diagram, data shown in magenta, grey and teal originates from three independent experiments with in total >8 analyzed images per condition. Error bars indicate mean ± STDEV.
f. Representative images of PomY-mCh in the absence or presence of 10 mM $MgCl_2$ (left) and in the presence of different KCl concentrations (4 µM PomY-mCh, 0 or 4% PEG8000). Top row: brightness and contrast comparable between images, set to 0-33; bottom row: brightness and contrast optimized for each image. The average separation factor in percent (right panel) is calculated as in (e). Data shown in magenta, grey and teal originates from three independent experiments with in total >8 analyzed images per condition. Error bars indicate mean ± STDEV.
g. Representative images of PomY-mCh in the presence of an increasing amount of 1,6-hexanediol (4 µM PomY-mCh, 0 or 4% PEG8000). Top row: brightness and contrast comparable between images, set to 0-43; bottom row: brightness and contrast optimized for each image. The average separation factor in percent (right panel) is calculated as in (e). Data shown in magenta, grey and teal originates from three independent experiments with in total 12 analyzed images per condition. Error bars indicate mean ± STDEV.

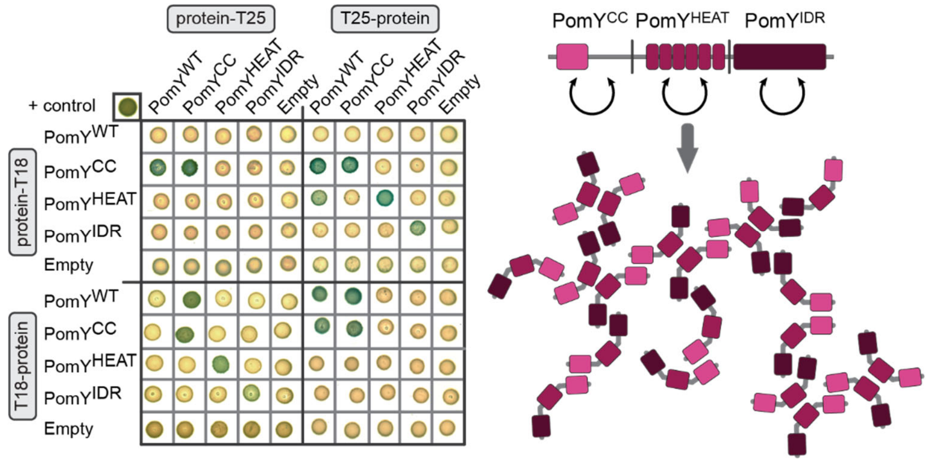

Supplementary Fig. 7. PomY self-interacts via multivalent interactions among all three PomY domains.
BACTH analysis of PomY and PomY domains. The indicated PomY domains (see Fig. 3a) were fused to the N- and the C-terminus of T18 and T25 as indicated. Blue and white colony colors indicate an interaction and no interaction, respectively. Positive control in upper left. The image shows representative results from three independent experiments. Upper right, schematic representation of the observed interactions indicated by arrows. Lower right, schematic representation of a possible interaction network between PomY molecules based on multivalent interactions. Colors of protein domains are as in the upper part.

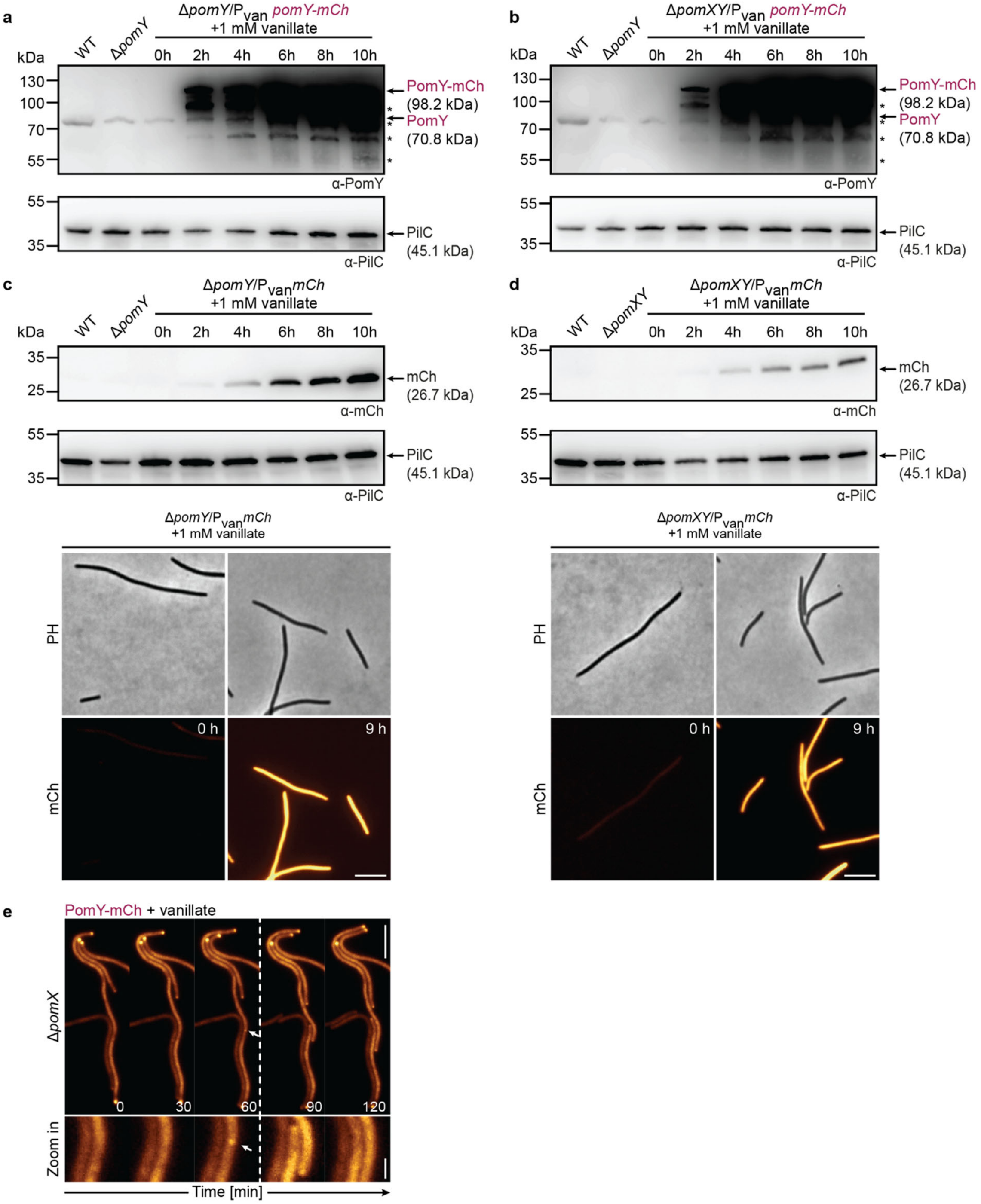

Supplementary Fig. 8. Analysis of strains extensively overproducing PomY-mCh.
a, b. Immunoblot analysis of PomY-mCh accumulation in total cell lysates of indicated strains. The same amount of protein was loaded per lane, separated by SDS-PAGE, blotted, and probed with specific α-PomY and α-PilC (loading control) antibodies. Molecular size markers are indicated on the left. Proteins with their calculated MW are indicated on the right. * indicates unspecific binding of the antibodies used. Overproduction of PomY-mCh was induced by the addition of 1 mM vanillate to cells in suspension at 0 h. The experiment was repeated three times, and representative results are shown. Note that PomY-mCh does not separate to run at the expected MW in SDS-PAGE, but instead run at a MW of ~120 kDa.

c, d. Immunoblot analysis of mCh in lysates of indicated strains (upper panel). Blots were done as in a but specific α-mCh antibodies were used. Lower panels, fluorescence microscopy of representative cells at the 0 h and 9 h time point. The experiment was repeated three times, and representative results are shown. Scale bar, 5 μm.
e. Fluorescence time-lapse microscopy of cells with a highly elevated PomY-mCh concentration during division. Cells were imaged as in Fig. 5d. White arrows indicate clusters immediately before cell division. Stippled lines indicate cell division events. The experiment was repeated four times, and representative cells are shown. Scale bars, 5 μm, 0.5 μm in the zoomed images.

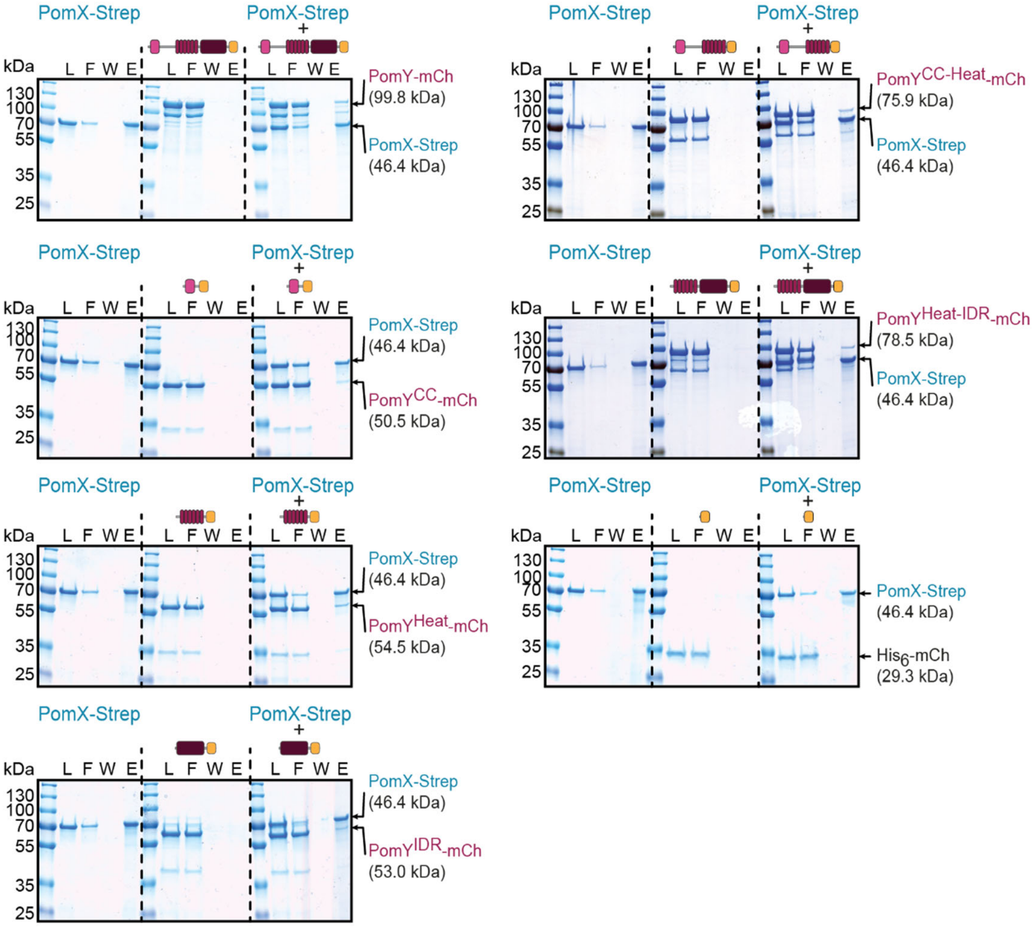

Supplementary Fig. 9. All three PomY domains interact with PomX.
In vitro pull-down experiments with PomX-Strep and PomY-mCh variants. Proteins were pulled-down using MagStrep XT beads with 10 μM proteins alone or in combination as indicated. Proteins from the load (L), flow-through (F), last wash (W), and elution fraction (E) were separated by SDS-PAGE and stained with Instant Blue. All samples in a panel were loaded on the same gel. Stippled lines are included for clarity. Experiments were repeated three times, and representative results are shown.

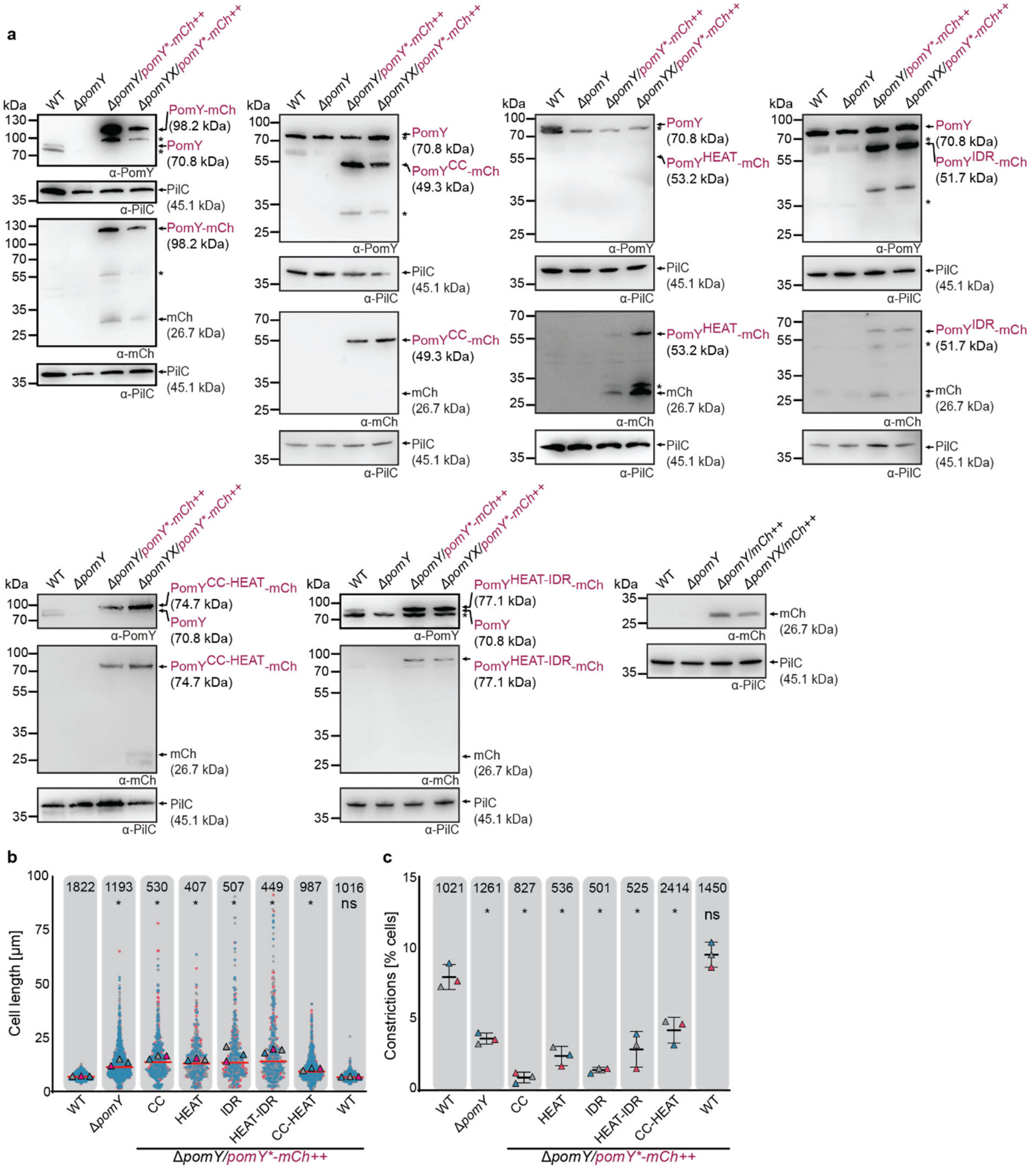

Supplementary Fig. 10. Analysis of truncated PomY-mCh variants.
a. Immunoblot analysis for the accumulation of the indicated PomY-mCh variants, and mCh in strains of the indicated genotype. The same amount of protein was loaded per lane, separated by SDS-PAGE, blotted, and probed with specific α-PomY, α-mCh antibodies and α-PilC (loading control) antibodies. Molecular size markers are indicated on the left. Proteins with their calculated MW are shown on the right. "*" indicates unspecific binding of the antibodies used. The experiments were repeated three times with similar results, and representative data are shown. ++ indicates that these genes were expressed from the *pilA* promoter. Note, that PomY-mCh and PomY^{IDR}-mCh do not separate to run at the expected MW in SDS-PAGE, but instead run at MW of ~120 kDa and 60 kDa, respectively.
b. Cell length distribution of strains expressing indicated PomY-mCh variants. ++ as in a. Data from three independent replicates are shown as magenta, grey, and teal. The means

are shown as a triangle in the relevant color. Red line shows the median. n are listed at the top. * indicates significant difference; ns, not significant in 2way ANOVA with multiple comparisons to WT. (*P*-values from left to right $P<0.0001$, $P<0.0001$, $P<0.0001$, $P<0.0001$, $P<0.0001$, $P<0.0001$, $P=0.9996$).

c. Constriction frequency analysis of cells of indicated genotypes. ++ as in a. Magenta, grey, and teal triangles show constrictions frequency in % of cells of three independent replicates. Black lines indicate the mean ± STDEV. n are listed at the top. * indicates significant difference; ns, not significant in 2way ANOVA with multiple comparisons to WT.(*P*-values from left to right $P=0.0001$, $P<0.0001$, $P<0.0001$, $P<0.0001$, $P<0.0001$, $P=0.0007$, $P=0.2714$).

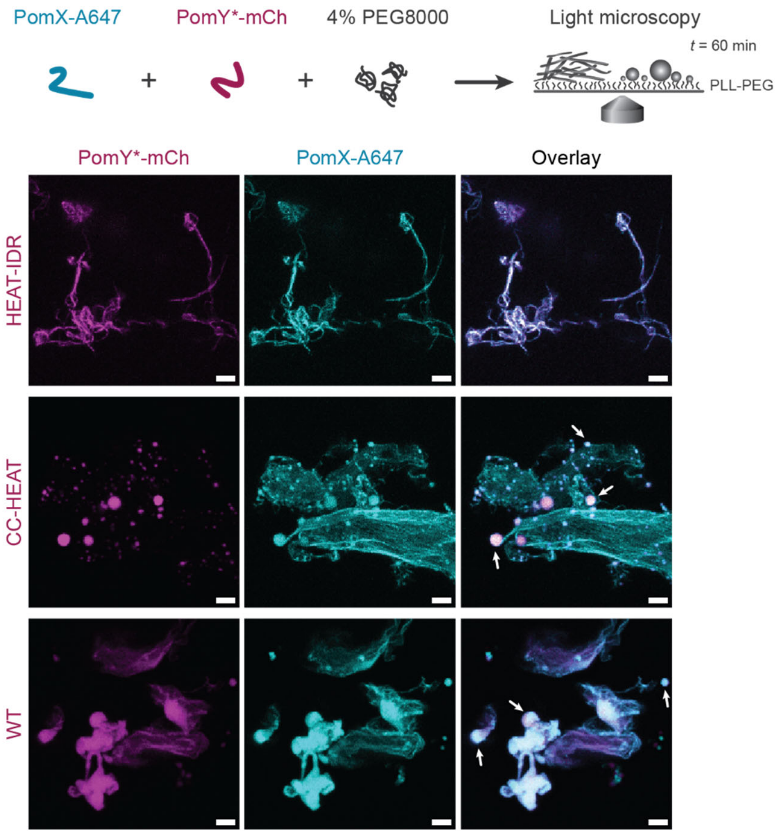

Supplementary Fig. 11. PomY$^{HEAT\text{-}IDR}$ coats PomX structures, but does not show formation of round condensates like PomY$^{CC\text{-}HEAT}$ or PomY-mCh.
Representative maximum intensity projections of confocal z-stacks of 4 µM PomX-A647 and 4 µM of the indicated PomY-mCh variant in the presence of 4% PEG8000. Experiment was performed three times. Scale bars, 5 µm.

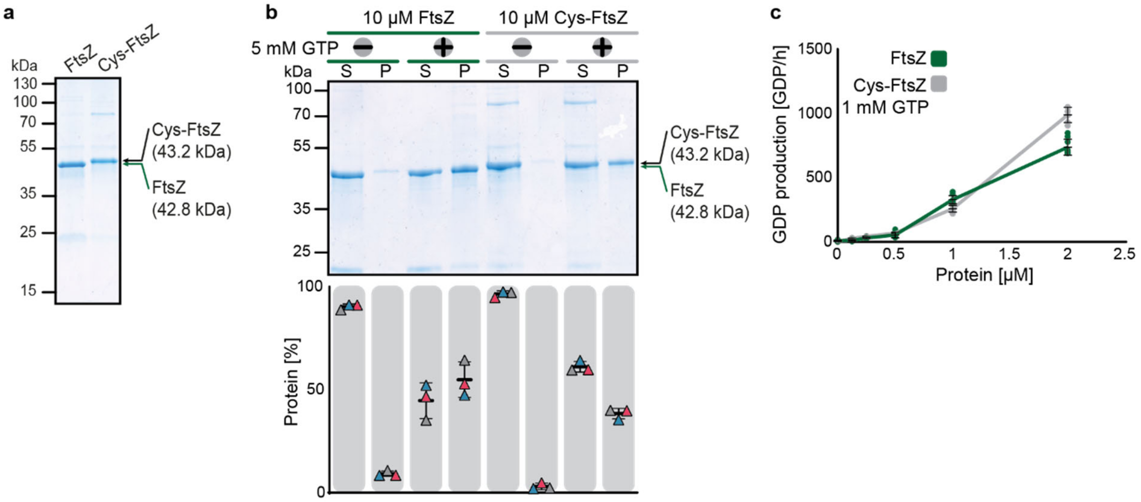

Supplementary Fig. 12. Characterization of Cys-FtsZ and A488-FtsZ.
a. Instant Blue-stained SDS-PAGE analysis of purified FtsZ and Cys-FtsZ. Molecular size markers are shown on the left and proteins with their molecular weight are shown on the right. 2 μg protein per lane were loaded. The experiment was repeated three times, and representative results are shown.
b. Instant Blue-stained SDS-PAGE of soluble and pellet fraction of 10 μM Cys-FtsZ (unlabeled) and FtsZ after application to high-speed centrifugation with and without the addition of 5 mM GTP (upper panel). Protein fractions of three independent replicates were quantified in % of total protein and plotted as colored triangles as the mean ± STDEV (lower panel).
c. Indicated concentrations of Cys-FtsZ (unlabeled) and FtsZ were assayed for their ability to hydrolyze GTP in the presence of 1 mM GTP. Green and grey dots indicate independent replicates. The mean ± STDEV are shown as black lines. n for FtsZ, 6; n for Cys-FtsZ, 9.

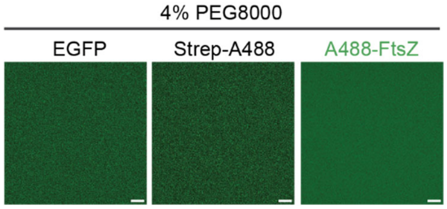

Supplementary Fig. 13. A488-FtsZ is enriched in PomY-mCh condensates, but does not form structures in presence of PEG8000.
Representative fluorescence microscopy images of 1 µM EGFP, Strep-A488 or A488-FtsZ in presence of 4% PEG8000. Experiment was performed five times. Scale bars, 5 µm

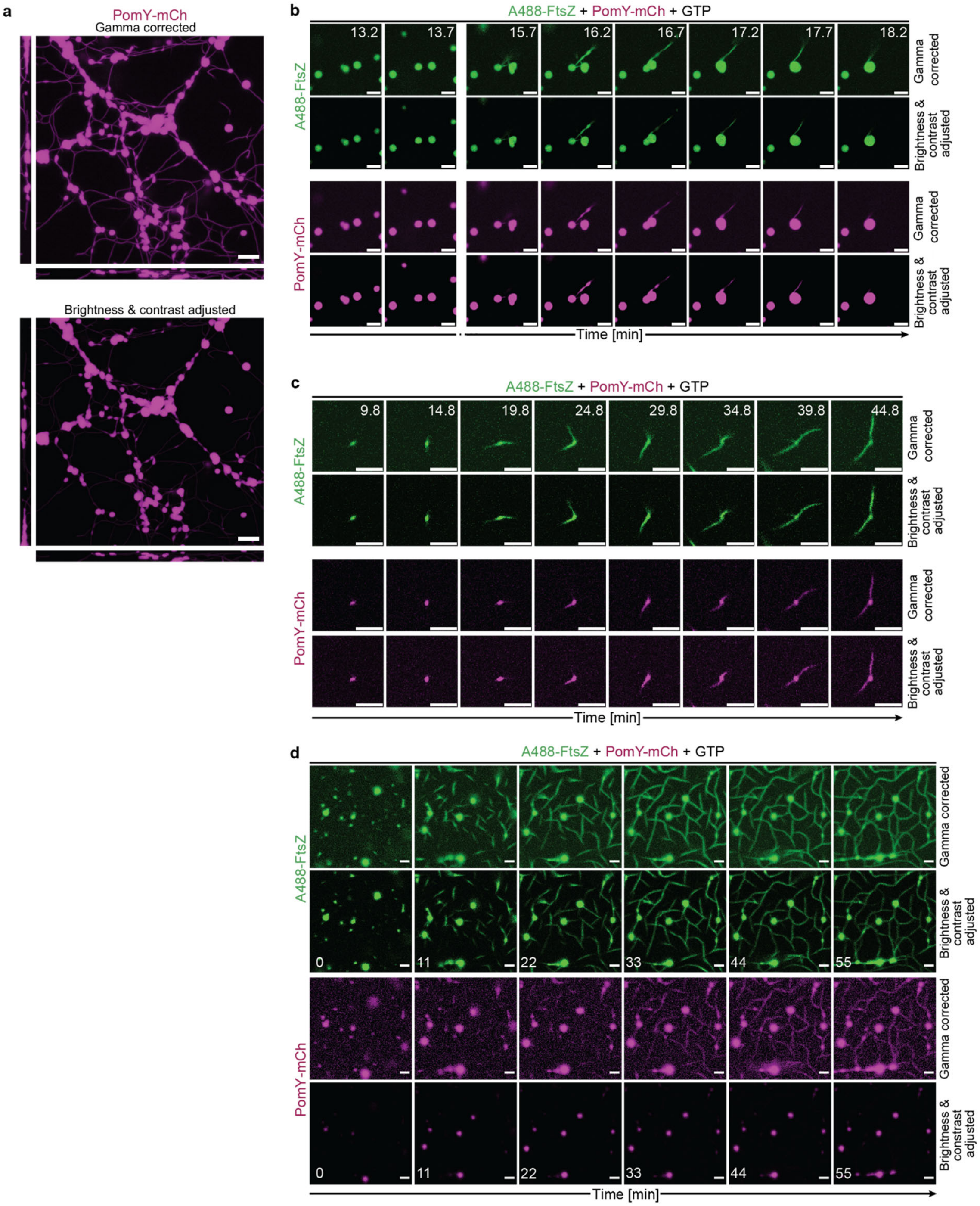

Supplementary Figure 14. Unaltered images for Figure 8.
a-c. Gamma-corrected and just brightness and contrast adjusted time-series as shown in Fig. 8e, f and g (same conditions and repeats), respectively. Scale bars, 5 µm.
d. Gamma-corrected and just brightness and contrast maximum intensity projection and z-projection as shown in Fig. 8h. Scale bars, 5 µm.

Supplementary Table 1. *M. xanthus* strains used in this study.

Strain	Genotype	Reference
SA4420	Δ*mglA*	[2]
SA4777	Δ*mglA*, Δ*pomX*	This study
SA4779	Δ*mglA*, Δ*pomY*	This study
SA4797	Δ*mglA*, Δ*pomX*/*attB*::P_{pomZ} *mCh-pomX* (pAH53)	[3]
SA7000	Δ*mglA*, Δ*pomY*/*attB*::P_{pilA} *pomY-mCh* (pDS7)	[3]
SA7045	Δ*mglA*, Δ*pomX*, Δ*pomY*	This study
SA7064	Δ*mglA*, Δ*pomY*/*attB*::P_{nat} *pomY-mCh* (pDS8)	This study
SA9734	Δ*mglA*; Δ*pomX*/*attB*::P_{pilA} *mCh-pomX* (pAH35)	This study
SA9764	Δ*mglA*, Δ*pomY*/*mxan18-19*::P_{van} *pomY-mCh* (pDS331)	This study
SA9768	Δ*mglA*, Δ*pomY*/*attB*::P_{pilA} $pomY^{CC}$-*mCh* (pPK29)	This study
SA9769	Δ*mglA*, Δ*pomX*, Δ*pomY*/*attB*::P_{pilA} *pomY-mCh* (pDS7)	This study
SA9770	Δ*mglA*, Δ*pomY*/*attB*::P_{pilA} $pomY^{CC-HEAT}$-*mCh* (pPK36)	This study
SA9772	Δ*mglA*, Δ*pomX*, Δ*pomY*/*attB*::P_{pilA} $pomY^{CC}$-*mCh* (pPK29)	This study
SA9779	Δ*mglA*, Δ*pomX*, Δ*pomY*/*attB*::P_{pilA} $pomY^{CC-HEAT}$-*mCh* (pPK36)	This study
SA9784	Δ*mglA*, Δ*pomX*, Δ*pomY*/*attB*::P_{nat} *pomY-mCh* (pDS8)	This study
SA9785	Δ*mglA*, Δ*pomX*, Δ*pomY*/*mxan18-19*::P_{van} *pomY-mCh* (pDS331)	This study
SA9794	Δ*mglA*, Δ*pomY*/*attB*::P_{pilA} *mCh* (pDS79)	This study
SA9795	Δ*mglA*, Δ*pomX*, Δ*pomY*/*attB*::P_{pilA} *mCh* (pDS79)	This study
SA9796	Δ*mglA*, Δ*pomY*/*mxan18-19*::P_{van} *mCh* (pAH225)	This study
SA9797	Δ*mglA*, Δ*pomX*, Δ*pomY*/*mxan18-19*::P_{van} *mCh* (pAH225)	This study
SA11802	Δ*mglA*, Δ*pomY*/*attB*::P_{pilA} $pomY^{HEAT}$-*mCh* (pPK45)	This study
SA11803	Δ*mglA*, Δ*pomX*, Δ*pomY*/*attB*::P_{pilA} $pomY^{HEAT}$-*mCh* (pPK45)	This study
SA11804	Δ*mglA*, Δ*pomY*/*attB*::P_{pilA} $pomY^{IDR}$-*mCh* (pPK46)	This study
SA11805	Δ*mglA*, Δ*pomX*, Δ*pomY*/*attB*::P_{pilA} $pomY^{IDR}$-*mCh* (pPK46)	This study
SA11806	Δ*mglA*, Δ*pomY*/*attB*::P_{pilA} $pomY^{HEAT-IDR}$-*mCh* (pPK47)	This study
SA11807	Δ*mglA*, Δ*pomX*, Δ*pomY*/*attB*::P_{pilA} $pomY^{HEAT-IDR}$-*mCh* (pPK47)	This study

Supplementary Table 2. Plasmids used in this study.

Plasmid	Genotype	Reference
pKA3	Overexpression of *pomZ-His$_6$* in *E. coli*; Amp^R	[4]
pKA28	P_{nat} *pomZ-mCh*; *attP*; Tc^R	[4]
pKA45	P_{pilA} *pomZ-mCh*; *attP*; Km^R	[3]
pKA70	Overexpression of *ftsZ* in *E.coli*; Amp^R	[4]
pDS3	Overexpression of *pomY-His$_6$* in *E. coli*; Km^R	[4]
pDS7	P_{pilA} *pomY-mCh*; *attP*; Km^R	[3]
pDS8	P_{nat} *pomY-mCh*; *attP*; Tet^R	[3]
pDS37	IPTG-dependent expression of *mCh-pomX* in *E. coli*; Km^R	[3]
pDS43	IPTG-dependent expression of *pomZ-mCh* in *E. coli*; Km^R	[3]
pDS46	IPTG-dependent expression of *pomY-yfp* in *E. coli*; Km^R	[3]
pDS79	P_{pilA} *mCh*; *attP*; Km^R	This study
pDS120	BACTH plasmid containing *pomY* (pUT18C); Amp^R	[1]
pDS121	BACTH plasmid containing *pomY* (pKT25); Km^R	This study
pDS122	BACTH plasmid containing *pomY* (pUT18); Amp^R	[1]
pDS123	BACTH plasmid containing *pomY* (pKNT25); Km^R	This study
pDS132	P_{pilA} *$pomY^{CC\text{-}HEAT}$-mCh; attP;* Km^R	This study
pDS261	BACTH plasmid containing *$pomY^{CC}$* (pUT18); Amp^R	This study
pDS262	BACTH plasmid containing *$pomY^{CC}$* (pUT18C); Amp^R	This study
pDS263	BACTH plasmid containing *$pomY^{CC}$* (pKNT25); Km^R	This study
pDS264	BACTH plasmid containing *$pomY^{CC}$* (pKT25); Km^R	This study
pDS265	BACTH plasmid containing *$pomY^{HEAT}$* (pUT18); Amp^R	This study
pDS266	BACTH plasmid containing *$pomY^{HEAT}$* (pUT18C); Amp^R	This study
pDS267	BACTH plasmid containing *$pomY^{HEAT}$* (pKNT25); Km^R	This study
pDS268	BACTH plasmid containing *$pomY^{HEAT}$* (pKT25); Km^R	This study
pDS269	BACTH plasmid containing *$pomY^{IDR}$* (pUT18); Amp^R	This study
pDS270	BACTH plasmid containing *$pomY^{IDR}$* (pUT18C); Amp^R	This study
pDS271	BACTH plasmid containing *$pomY^{IDR}$* (pKNT25); Km^R	This study
pDS272	BACTH plasmid containing *$pomY^{IDR}$* (pKT25); Km^R	This study
pDS331	P_{van} *pomY-mCh*; *mxan18-19*; Km^R	This study

pEMR1	Overexpression of *pomY-His$_6$* in *E. coli*; Km^R	[1]
pEMR3	Overexpression of *pomX-His$_6$* in *E. coli*; Km^R	[3]
pAH14	Overexpression of His$_6$-*mCh;* Amp^R	This study
pAH35	P$_{pilA}$ *mCh-pomX; attP;* Km^R	[3]
pAH53	P$_{mxan0635}$ *mCh-pomX; attP;* Km^R	[3]
pAH180	Overexpression of Cys-*ftsZ* in *E. coli*; Km^R	This study
pAH187	Overexpression of *pomY$^{CC-HEAT}$–mCh*-His$_6$ in *E. coli*; Km^R	This study
pAH194	Overexpression of *pomY-mCh*-His$_6$ in *E. coli*; Km^R	This study
pAH195	Overexpression of *pomY$^{HEAT-IDR}$-mCh*-His$_6$ in *E. coli*; Km^R	This study
pAH196	Overexpression of *pomY^{CC}-mCh*-His$_6$ in *E. coli*; Km^R	This study
pAH197	Overexpression of *pomY^{HEAT}–mCh*-His$_6$ in *E. coli*; Km^R	This study
pAH198	Overexpression of *pomY^{IDR}-mCh*-His$_6$ in *E. coli*; Km^R	This study
pAH205	Overexpression of *pomX-Strep* in *E. coli*; Km^R	This study
pAH225	P$_{van}$ *mCh; mxan18-19*; Km^R	This study
pPK29	P$_{pilA}$ *pomY^{CC}-mCh; attP;* Km^R	This study
pPK30	P$_{pilA}$ *pomY^{HEAT}-mCh; attP;* Km^R	This study
pPK36	P$_{pilA}$ *pomY$^{CC-HEAT}$-mCh; attP;* Km^R	This study
pPK45	P$_{pilA}$ *pomY^{HEAT}-mCh; attP;* Km^R	This study
pPK46	P$_{pilA}$ *pomY^{IDR}-mCh; attP;* Km^R	This study
pPK47	P$_{pilA}$ *pomY$^{HEAT-IDR}$-mCh; attP;* Km^R	This study
pSL16	Construct for in-frame deletion of *mglA* (*mxan1925*)	[2]
pMR3690	Plasmid for vanillate inducible gene expression in *M.xanthus; mxan18-19;* Km^R	[5]
pSWU30-*pomY$^{CC-HEAT}$-mCh*	P*pilA pomY$^{CC-HEAT}$*-mCh; *attP*; Tet^R	This study
pBR35	Overexpression of His$_6$-*pomZ-mCh* in *E. coli*; Amp^R	This study
pBR36	Overexpression of His$_6$-*mCh-pomX* in *E. coli*; Km^R	This study
pBR38	Overexpression of *pomY-yfp*-His$_6$ in *E. coli*; Km^R	This study
pBR39	Overexpression of *pomX*-His$_6$-Cys in *E. coli*; Km^R	This study
pBR72	Overexpression of *pomY-mCh*-His$_6$ in *E. coli*; Km^R	This study
pUT18	BACTH plasmid; Amp^R	[6]
pUT18C	BACTH plasmid; Amp^R	[6]
pKT25	BACTH plasmid; Km^R	[6]
pKNT25	BACTH plasmid; Km^R	[6]

pKT25-zip	BACTH plasmid containing leucine zipper of GCN4; Amp^R	[6]
pUT18C-zip	BACTH plasmid containing leucine zipper of GCN4; Km^R	[6]

Supplementary Table 3. Oligonucleotides used in this work.

Oligonucleotide	Sequence 5'-3'
mCherry fwd Start XbaI	GCGTCTAGAATGGTGAGCAAGGGCGAGGAG
mCherry stop rev HindIII	GGGAAGCTTTTACTTGTACAGCTCGTC
MXAN_0634-6	GCGTCTAGAGTGAGCGACGAGCGTCCG
DS1	CCGGAATTCCGACGAGCAGTTGAGCACCAG
DS2	GCGAGATCTGGCGGAGCCCCGCGCCCGCACAGGC
DS14	GCGCATATGAGCGACGAGCGTCCG
DS40	GCGGAATTCTTACTTGTACAGCTCGTC
KA477	GCGGGATCCCATGGTGAGCAAGGGCGAGGAG
KA478	GCCAAGCTTTCATTACTTGTACAGCTCGTCCAT
AH159	GGAATTCCATATGGGCAAGTGCAAGGGCTCCGGCGAC CAGTTCGATCAGAACAAGC
KA500	GCGGGATCCTTATTACGGCAGTTCCGTCTGGC
NdeI PomY fwd	GGAATTCCATATGAGCGACGAGCGTCCGGAC
DS274	GCGAAGCTTCTTGTACAGCTCGTCCATGC
DS12	GCGCATATGGGACGGTTGGGCGTGAGC
DS271	GCGCATATGCTGGAGCAGCCTCGTCCG
DS278	GCGTCATGAAGAAAGCCTTTGAACAGAACG
DS276	GCGAAGCTTACTTCTCGAACTGTGGGTGACTCCAGCGC ACCGTGGCCTGAC
mCherry fwd NdeI	GCGCATATGGTGAGCAAGGGCGAGGAGG
CC(nat) linker rev BglII	CGCAGATCTGGCGGACGCCTGCAGGAAGCGCTGCG
17 HEAT(nat) XbaI fwd	GCGTCTAGAGTGGGACGGTTGGGCGTGAGCGC
18 HEAT(nat) linker rev BglII	CGCAGATCTGGCGGACCGCGCCCGCACAGGCTTC
DS4	GCGTCTAGAGTGAGCGACGAGCGTCCG
27 HEAT fwd	GCGTCTAGAGGACGGTTGGGCGTGAGCGC
FM4	GCGTCTAGACTGGAGCAGCCTCGTCCG
BR68	GGCCTGCTGGGTGCC
BR70	ATGGACGAGCTGTACAAGTGATGAAAGCTTGCGGCCG
BR64	CTTGTACAGCTCGTCCATGCC
BR69	CCGTCGCCCAGACGAAG
BR71	TCCGCGGGTCTGGAAGTTCTGTTCCAGGGGCCCAGCAA GGGCGAGGAG
BR72	GTGATGGTGATGGTGATGTTTCATGGTATATCTCCTTATT AAAGTTAAACAAA
BR62	CATGGACGAGCTGTACAAGAAGCTTGCGGCCGCAC
BR75	CATATGTATATCTCCTTCTTAAAGTTAAACAAAATTATTTC TAGAG
BR77	GAAGGAGATATACATATGAGCGACGAGCGTCCG
BR87	GTGGTGGTGGTGGTGGTG
BR153	GGTGGTTCTGGTTGCGGTAGCGGCAGCGGTAGCTGAGA TCCGGCTGCTAACAAAGC
BR185	CTCCAGAACCTCCTCCACCAGCGGCGAAGTATTTGTGCC
BR184	GGTGGAGGAGGTTCTGGAGGCGGTGGAAGTGGTGGCGG AGGTAGCATGGTGAGCAAGGGCGAG
Mxan0634 fwd BACTH	GCGTCTAGAGGTGAGCGACGAGCGTCCG
Mxan_0634-17 pomY-rev	GCGGGTACCCGAGCGGCGAAGTATTTGTG
Mxan0634 Coil KpnI BACTH	GCGGGTACCCGCGCCTGCAGGAAGCGCTG
Mxan0634 HEAT XbaI BACTH	GCGTCTAGAGATGGGACGGTTGGGCGTGAGC

Mxan0634 HEAT KpnI BACTH	GCGGGATCCCGCCGCGCCCGCACAGGC
Mxan0634 Pro XbaI BACTH	GCGTCTAGAGATGCTGGAGCAGCCTCGTCCG

Supplementary references

1. Schumacher, D., Harms, A., Bergeler, S., Frey, E. & Søgaard-Andersen, L. PomX, a ParA/MinD ATPase activating protein, is a triple regulator of cell division in *Myxococcus xanthus*. *Elife* **10**, e66160 (2021).

2. Miertzschke, M. *et al.* Structural analysis of the Ras-like G protein MglA and its cognate GAP MglB and implications for bacterial polarity. *EMBO J.* **30**, 4185-4197 (2011).

3. Schumacher, D. *et al.* The PomXYZ proteins self-organize on the bacterial nucleoid to stimulate cell division. *Dev. Cell* **41**, 299-314 e213 (2017).

4. Treuner-Lange, A. *et al.* PomZ, a ParA-like protein, regulates Z-ring formation and cell division in *Myxococcus xanthus*. *Mol. Microbiol.* **87**, 235-253 (2013).

5. Iniesta, A.A., Garcia-Heras, F., Abellon-Ruiz, J., Gallego-Garcia, A. & Elias-Arnanz, M. Two systems for conditional gene expression in *Myxococcus xanthus* inducible by isopropyl-beta-D-thiogalactopyranoside or vanillate. *J. Bacteriol.* **194**, 5875-5885 (2012).

6. Karimova, G., Pidoux, J., Ullmann, A. & Ladant, D. A bacterial two-hybrid system based on a reconstituted signal transduction pathway. *Proc. Natl. Acad. Sci. USA* **95**, 5752-5756 (1998).
