# Supplementary material for: Biomolecular condensate drives polymerization and bundling of the bacterial tubulin FtsZ to regulate cell division
Source: Nat Commun. 2023 Jun 28;14:3825. doi: 10.1038/s41467-023-39513-2 (PMC10307791; doi:10.1038/s41467-023-39513-2)
Supplement: Supplementary file 2 — Description of Additional Supplementary Files [file 41467_2023_39513_MOESM2_ESM.pdf]

Description of Additional Supplementary Files

Supplementary Movie 1: PomY-mCh condensates undergo fusion and relax into spherical shapes. Two representative time-series of PomY-mCh condensates undergoing fusion (conditions: 4 µM PomY-mCh, 4% PEG8000 w/v). White insets represent the location of the snapshots shown Fig. 3h. Scale bars, 5 µm.

Supplementary Movie 2: PomY-mCh condensates wet PomX-A488 filaments and deform upon contact. Time-series of PomY-mCh and PomX-A488 in the presence of 4% PEG8000 as shown in the snapshots in Fig. 4e. Scale bars, 5 µm. Note that in this recording, the PomY-mCh condensates do not stimulate PomX-A488 filament bundle formation, instead the PomX-A488 filament bundle sediments from the solution into the focal plane for imaging.

Supplementary Movie 3: Dynamics of A488-FtsZ filament bundle formation and PomY-mCh condensate fusion/relaxation events.
Time-lapse recording of A488-FtsZ in the presence of PomY-mCh, PEG8000 and GTP. Images were acquired close to the coverslip surface. Representative frames of two movies are shown in Fig. 8f and g. The images have been gamma-corrected for better visualization. Scale bars, 5 µm.

Supplementary Movie 4: A488-FtsZ filament bundles emerge from PomY-mCh condensates and align and fuse to give rise to filamentous networks.
Time-series of A488-FtsZ in the presence of PomY-mCh, PEG8000 and GTP. Images were acquired close to the coverslip surface. White inset represents the location of the snapshots shown Fig. 8h and Supplementary Fig. 14. The images have been gamma-corrected for better visualization. Scale bars, 5 µm.
