# Supplementary material for: Biomolecular condensate drives polymerization and bundling of the bacterial tubulin FtsZ to regulate cell division
Source: Nat Commun. 2023 Jun 28;14:3825. doi: 10.1038/s41467-023-39513-2 (PMC10307791; doi:10.1038/s41467-023-39513-2)
Supplement: Supplementary file 7 — Reporting Summary [file 41467_2023_39513_MOESM7_ESM.pdf]

nature portfolio

Corresponding author(s): Beatrice Ramm
Dominik Schumacher
Petra Schwille
Lotte Søgaard-Andersen

Last updated by author(s): May 18, 2023

Reporting Summary

Nature Portfolio wishes to improve the reproducibility of the work that we publish. This form provides structure for consistency and transparency in reporting. For further information on Nature Portfolio policies, see our Editorial Policies and the Editorial Policy Checklist.

Statistics

For all statistical analyses, confirm that the following items are present in the figure legend, table legend, main text, or Methods section.

n/a	Confirmed	
☐	☒	The exact sample size (n) for each experimental group/condition, given as a discrete number and unit of measurement
☐	☒	A statement on whether measurements were taken from distinct samples or whether the same sample was measured repeatedly
☐	☒	The statistical test(s) used AND whether they are one- or two-sided<br>*Only common tests should be described solely by name; describe more complex techniques in the Methods section.*
☒	☐	A description of all covariates tested
☒	☐	A description of any assumptions or corrections, such as tests of normality and adjustment for multiple comparisons
☐	☒	A full description of the statistical parameters including central tendency (e.g. means) or other basic estimates (e.g. regression coefficient) AND variation (e.g. standard deviation) or associated estimates of uncertainty (e.g. confidence intervals)
☐	☒	For null hypothesis testing, the test statistic (e.g. F, t, r) with confidence intervals, effect sizes, degrees of freedom and P value noted<br>*Give P values as exact values whenever suitable.*
☒	☐	For Bayesian analysis, information on the choice of priors and Markov chain Monte Carlo settings
☒	☐	For hierarchical and complex designs, identification of the appropriate level for tests and full reporting of outcomes
☒	☐	Estimates of effect sizes (e.g. Cohen's d, Pearson's r), indicating how they were calculated

Our web collection on statistics for biologists contains articles on many of the points above.

Software and code

Policy information about availability of computer code

Data collection	In vitro fluorescence imaging and Structured Illumination Microscopy was performed with Zen Black (3.0 SR) (Zeiss). Bacterial fluorescence microscopy was otherwise performed with LAS-X (3.7.6.25997) (Leica).
Data analysis	Data analysis of all bacterial fluorescence microscopy was performed with MatLab (R2018a) and a self-written code (available at https://github.com/SBergeler/ImageAnalysisMyxo) or Metamorph (7.10.2.240), as described in the material and methods section. Image analysis of in vitro fluorescence microscopy was performed with Fiji (v1.53q), Oufti (v1.0) and MatLab (R2018a). Statistical analysis of cell biological data was performed with Graph Pad Prism 9 (9.0.2. (161)).

For manuscripts utilizing custom algorithms or software that are central to the research but not yet described in published literature, software must be made available to editors and reviewers. We strongly encourage code deposition in a community repository (e.g. GitHub). See the Nature Portfolio guidelines for submitting code & software for further information.

Data

Policy information about availability of data

All manuscripts must include a data availability statement. This statement should provide the following information, where applicable:

- Accession codes, unique identifiers, or web links for publicly available datasets
- A description of any restrictions on data availability
- For clinical datasets or third party data, please ensure that the statement adheres to our policy

All the data supporting the findings of this manuscript are available within the article, its Supplementary Information file or in the source data file. The source data for all figures and supplementary figures are provided in the source data file including uncropped and unprocessed scans of western blots and SDS-PAGE images.

Human research participants

Policy information about studies involving human research participants and Sex and Gender in Research.

Reporting on sex and gender	No human research participants are included in this research.
Population characteristics	See above
Recruitment	See above
Ethics oversight	See above

Note that full information on the approval of the study protocol must also be provided in the manuscript.

Field-specific reporting

Please select the one below that is the best fit for your research. If you are not sure, read the appropriate sections before making your selection.

[x] Life sciences [ ] Behavioural & social sciences [ ] Ecological, evolutionary & environmental sciences

For a reference copy of the document with all sections, see nature.com/documents/nr-reporting-summary-flat.pdf

Life sciences study design

All studies must disclose on these points even when the disclosure is negative.

Sample size	No statistical methods were used to predetermine the sample size. The sample size was determined based on our expertise in bacterial cell biology. Generally, three biological replicates with a high number of cells (n) were used for the image analysis of fluorescence pictures. Similarly, we decided that based on our own experience, for biochemical experiments, several independent protein purifications served as biological replicates. The sample size used for our analyses provides enough single cell measurements to allow robust detection of phenotypes and to draw main conclusions made in this study.
Data exclusions	Images were only excluded from data analysis of in vitro experiments when obvious protein aggregates made data analysis impossible.
Replication	Data shown for fluorescence microscopy, live-cell imaging, immunoblot experiments, sedimentation assays and in vitro pull-down experiments were obtained in at least three independent experiments with similar results if not stated differently.
Randomization	The experiments were not randomized, since there was no allocation into subgroups.
Blinding	The experiments were not blinded, since there was no allocation into subgroups.

Reporting for specific materials, systems and methods

We require information from authors about some types of materials, experimental systems and methods used in many studies. Here, indicate whether each material, system or method listed is relevant to your study. If you are not sure if a list item applies to your research, read the appropriate section before selecting a response.

Materials & experimental systems

n/a	Involved in the study
☐	☒ Antibodies
☒	☐ Eukaryotic cell lines
☒	☐ Palaeontology and archaeology
☒	☐ Animals and other organisms
☒	☐ Clinical data
☒	☐ Dual use research of concern

Methods

n/a	Involved in the study
☒	☐ ChIP-seq
☒	☐ Flow cytometry
☒	☐ MRI-based neuroimaging

Antibodies

Antibodies used

All antibodies used in this study are described in the Methods section. For western blot analysis of M.xanthus samples the following rabbit polyclonal antibodies were used: α-PomX and α-PomY in a 1:10000 dilution (Schumacher et al 2017); α-PilC in a 1:3000 dilution (Bulyha et al., 2009).
Polyclonal, rabbit α-mCherry antibodies in a 1:10000 dilution (BioVision; 5993-100) were used to detect mCherry-tagged proteins.
As secondary antibodies goat, anti-rabbit immunoglobulin G peroxidase conjugate antibodies were used in a 1:25000 dilution (Sigma-Aldrich, A8275).

Validation

α-PomX and α-PomY antibodies are described in Schumacher et al., 2017; https://doi.org/10.1016/j.devcel.2017.04.011
α-PilC antibodies are described in Bulyha et al., 2008; DOI: 10.1111/j.1365-2958.2009.06891.x

The web addresses of commercially available antibodies are as follows:
α-mCherry antibodies (https://www.biovision.com/mcherry-antibody.html)
goat, anti-rabbit immunoglobulin G peroxidase conjugate antibodies (https://www.sigmaaldrich.com/DE/de/product/sigma/a8275)
